# Giant Inguino-Scrotal Hernia With Loss of Domain: Surgical Report and Literature Review

**DOI:** 10.7759/cureus.74599

**Published:** 2024-11-27

**Authors:** Alyssa Koller, Jose Oberholzer, Fabian Rössler

**Affiliations:** 1 Surgery and Transplantation, Universitätsspital Zürich, Zürich, CHE

**Keywords:** abdominal component separation technique, giant inguinal hernia, inguinal hernia repair, loss of abdominal domain, open hernia surgery

## Abstract

The surgical repair of giant inguinal hernias with loss of domain, defined as the relocation of the majority of the intestine into the hernia sac, poses a significant challenge. In the majority of cases, a combination of different surgical techniques with the placement of multiple meshes is necessary to achieve reduction of such complex hernias. The reduction of chronic giant hernias can increase the risk of abdominal compartment syndrome or cardiopulmonary complications. This case study presents a rare and complex case of a patient with a chronic giant inguinal hernia, in which almost the entire intestine was herniated, involving the scrotum and reaching mid thigh. The reduction of the hernia was achieved by a combined open transabdominal and inguinal approach, utilizing the abdominal component separation technique and multiple preperitoneal mesh placements. This multimodal approach resulted in optimal outcomes in terms of cosmesis, functionality, and abdominal wall integrity.

## Introduction

Inguinal hernias (IH) are a prevalent condition, with IH repair representing one of the most common surgical procedures in the Western world. A variety of surgical techniques have been described, encompassing open, laparoscopic, and robotic-assisted repairs. Currently, the majority of IH repairs are conducted with the placement of a mesh. However, giant inguino-scrotal hernias (GIH) are uncommon and often chronic, exhibiting slow growth over several years. If left untreated for an extended period, a condition known as "loss of domain" (LOD) may develop, characterized by the enlargement of the hernia defect to a point where a considerable portion of the viscera protrudes into the hernia sac [1]. To date, a number of definitions of a GIH have been proposed. The most commonly accepted definition includes the extent of the sac below the mid-inner thigh when the patient is in an upright position [2]. Other definitions include an anterior-posterior diameter of the hernia sac of at least 30 cm, or a latero-lateral diameter of approximately 50 cm, with no reduction for more than 10 years [3]. The clinical manifestations of GIH are highly variable. However, the majority of cases are asymptomatic and associated with long-term neglect by the patient, which facilitates the chronic progression of the disease. In rare instances, complications may arise, such as intestinal strangulation, urinary symptoms, or ipsilateral testicular atrophy.

Over the past few decades, there have been notable developments in the field of IH repair techniques and abdominal wall surgery. The concept of tension-free repair represents a significant advancement in this area [4]. A number of open and minimally invasive techniques for the repair of IH and ventral hernias have been successfully introduced. Nevertheless, a standardized surgical approach for the repair of GIH has yet to be established, and large-scale comparative studies are lacking. GIH with LOD presents a particular technical challenge and is associated with an increased risk of postoperative complications. Due to the size and location of the hernia, special considerations for the repair of the abdominal wall must be taken into account, and strategies differ from those employed in simpler ventral hernia repairs.

Firstly, the reduction of hernia contents to the abdominal cavity can result in the development of excessive intra-abdominal pressure, which may subsequently lead to the onset of severe cardiorespiratory (CR) complications and even abdominal compartment syndrome (ACS). This must be taken into account prior to repositioning of GIH, and a combination of different surgical accesses may be necessary. In order to overcome the issue of limited space, a number of management strategies have been proposed, including resection, abdominal wall component separation, preoperative pneumoperitoneum, and muscle relaxation [5-8].

Secondly, a number of different surgical techniques may be necessary to achieve a complete reduction of the hernia and create sufficient space. This increases the risk of wound infection and wound healing disorders, particularly when the wound area is extended, especially in combination with inguinal or scrotal incisions. Thirdly, due to the typically chronic course of GIH, anatomical structures may undergo alteration, and severe adhesions of viscera within the hernia sac may complicate surgical repair and hernia repositioning. Fourthly, the scrotum is frequently affected and typically requires partial resection and reconstruction to avoid wound complications and improve the cosmetic outcome.

This case study presents a patient suffering from GIH with excessive LOD, in which the majority of the intestine was herniated. The surgical repair was complicated by the fixed intestinal loops and ipsilateral testicular atrophy, necessitating combined transabdominal and inguinal surgical accesses. Three meshes were placed in a preperitoneal position to successfully close the giant hernia, and abdominal closure was achieved by components separation technique to overcome the space problem. The postoperative course was without major complications, and the long-term results were excellent in terms of function, cosmesis, and prevention of recurrence.

## Case presentation

**Figure 1:**
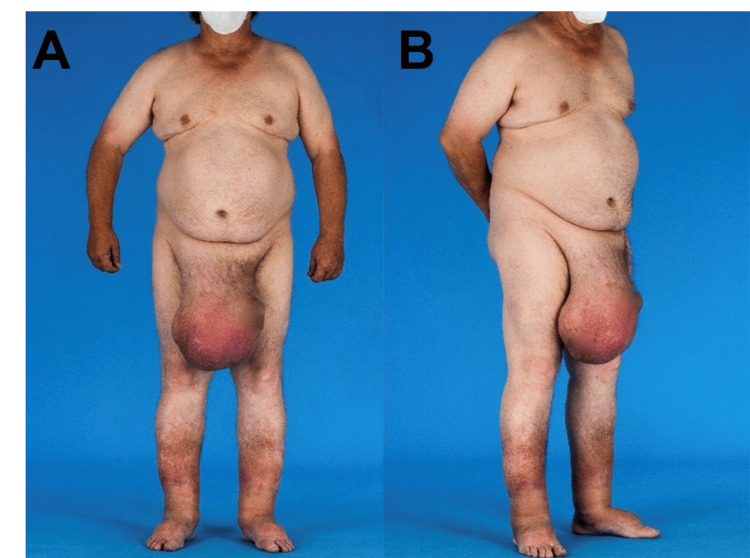
Preoperative image of the giant inguino-scrotal hernia with loss of domain. (A) Preoperative image front view and (B) preoperative image lateral view.

Our patient, a 68-year-old male, presented with a large inguino-scrotal hernia that was not reducible. Substantial enlargement of the swelling had been observed for over a decade and had been gradually increasing in size. In the six months preceding the patient's initial presentation, the swelling had increased significantly in size and had become symptomatic. Additionally, the patient presented with obesity (BMI 35 kg/m²), diabetes mellitus type II, arterial and pulmonary-arterial hypertension, and obstructive sleep apnea with the need for continuous positive airway pressure (CPAP) therapy. There was no previous history of abdominal or inguinal surgery. The swelling significantly restricted the patient's mobility and caused abdominal discomfort. However, the patient did not experience pain or constipation. The diameter of the hernia was approximately 35x30 cm, extending just below the patient's right knee. Additionally, there were indications of chronic alterations in the skin and scrotum, including thickening. The penis was not visible, and the testicles were no longer discernible. Additionally, an umbilical hernia and a smaller left inguinal hernia were present, both of which were asymptomatic and contained only fatty tissue. A large right-sided hydrocele testis was also observed (Figures 1A, 1B).

**Figure 2:**
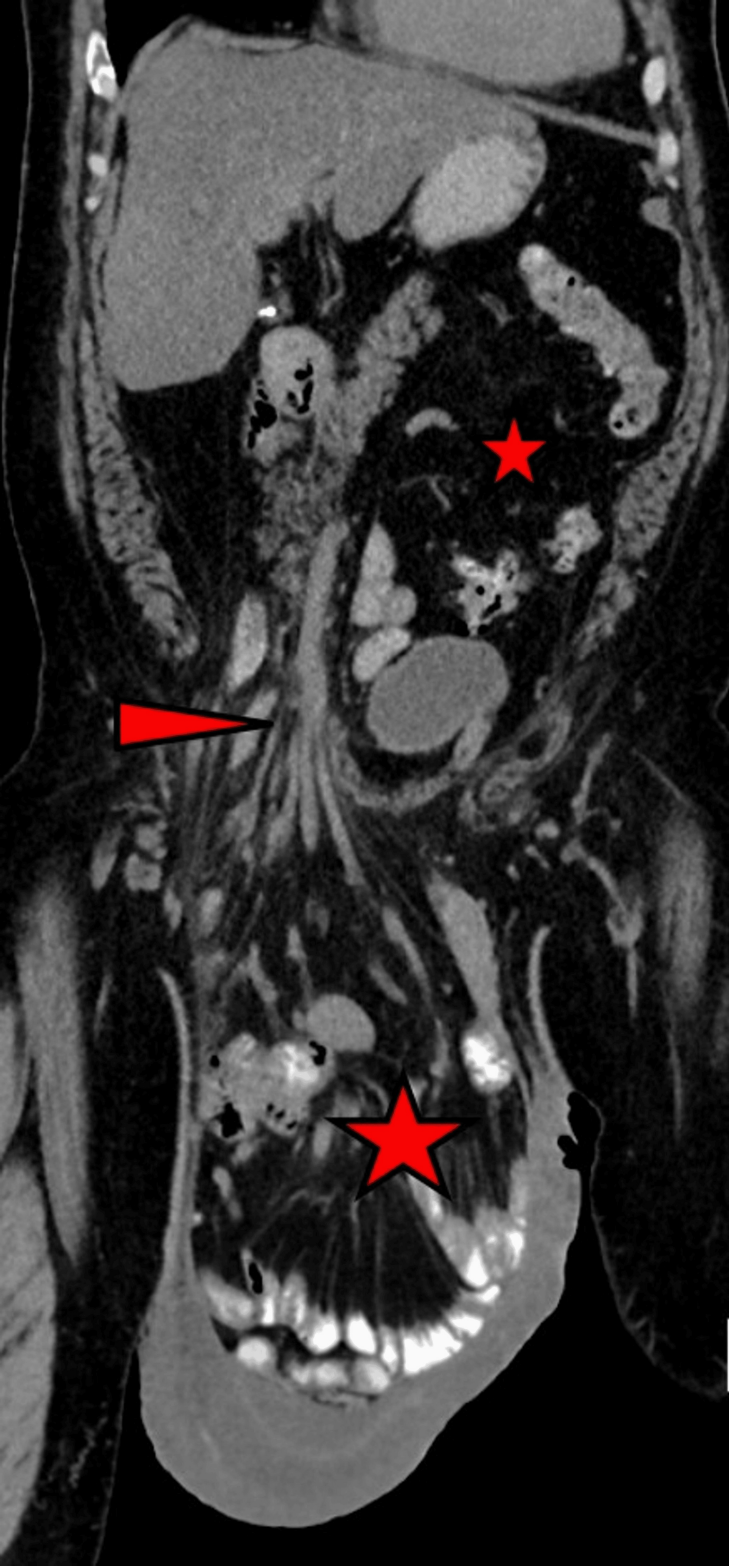
Preoperative computed tomography. Arrowhead shows inguinal canal with herniated intestine and mesenteric root; small star shows abdominal cavity, with almost no small intestine left; and large star shows small intestine in the giant hernia sac.

**Figure 3:**
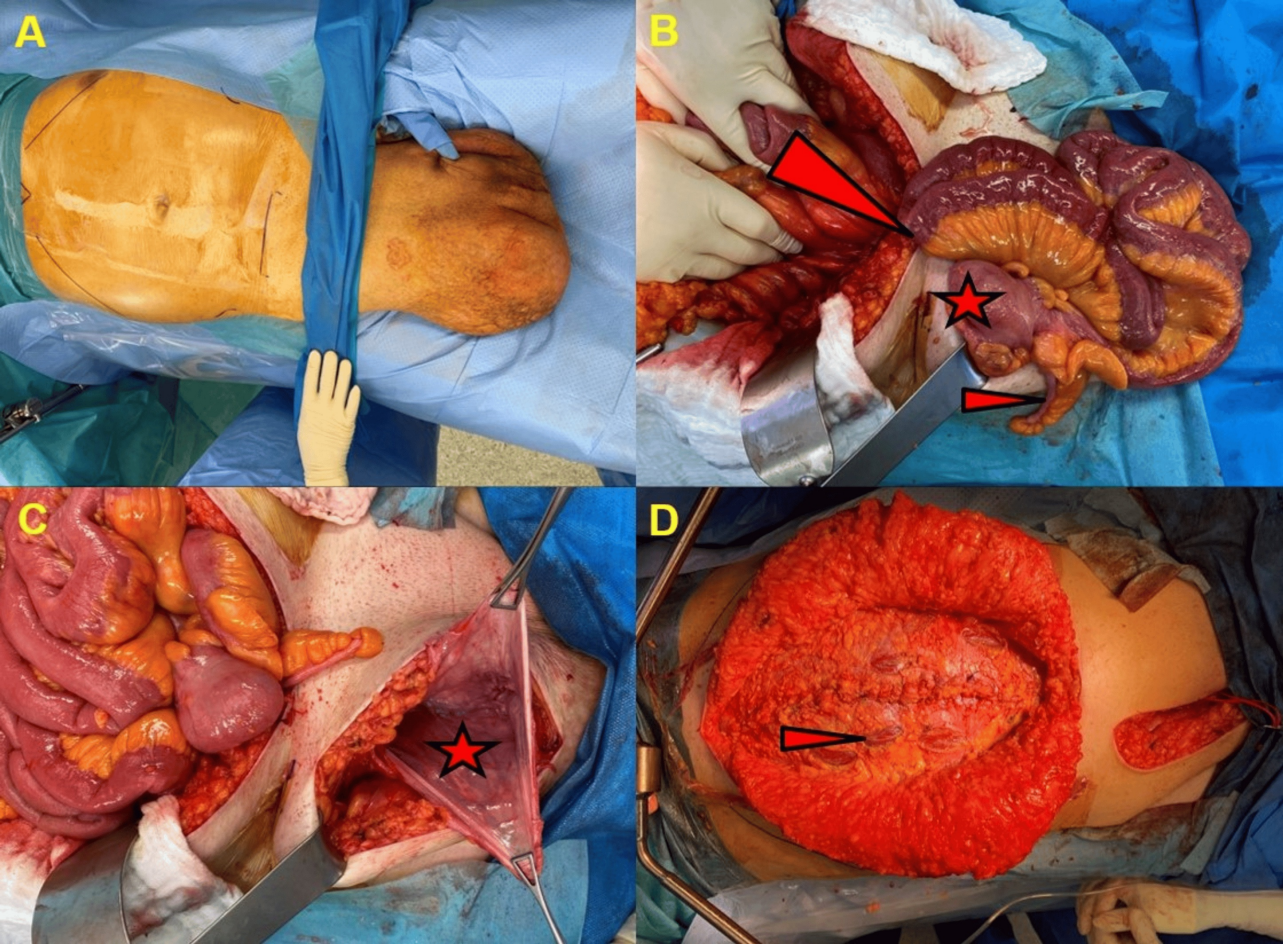
Images of different stages of the operation. (A) Preoperative setting before incision; (B) manual reposition of herniated small intestine, cecum, and appendix vermiformis (large arrowhead shows inguinal canal with herniated intestine, star shows herniated cecum, small arrowhead shows herniated appendix vermiformis); (C) after complete hernia reduction (star shows empty hernia sac on proximal right thigh); and (D) situs after complete closure of the abdomen (small arrowhead shows longitudinal incisions along the ventral fascia for tension reduction).

A CT scan revealed a massive LOD, containing almost the entire small intestine and part of the right colon (Figure 2). There were no clinically or radiologically evident signs of incarceration. Given the considerable dimensions of the hernia, a midline laparotomy was deemed the optimal approach. This entailed a component separation technique and the placement of three meshes in a preperitoneal position. This approach was selected to combine inguinal and abdominal wall reinforcement and to prevent space issues following hernia reduction. This surgical technique is analogous to an extended bilateral Stoppa procedure. Furthermore, a preoperative botulinum injection was administered three weeks prior to the surgery to facilitate abdominal expansion. A total of 300 IU of botulinum toxin A (BTX) was injected into the muscle layers on both sides. The intraoperative images are shown in Figures 3A-3D.

Following midline laparotomy, adhesions around the umbilical hernia and the greater omentum were successfully resolved. No evidence of intestinal strangulation was observed. As anticipated based on the CT imaging, the majority of the small intestine and a portion of the right colon were herniated through the inguinal canal and could not be reduced. Consequently, a longitudinal incision was made, commencing just below the right inguinal ligament and extending to the midpoint of the right thigh. At this point, the large hernia sac was separated, revealing the entire small intestine, including the cecum and the appendix vermiformis. Although the hernia passage itself was rather narrow, there were no signs of intestinal strangulation or constipation. In order to create more space in the abdomen, the main part of the greater omentum was resected. By applying pressure from both sides, the entire hernia contents could be successfully reduced back into the abdominal cavity. Subsequently, the large hernia sac was completely resected after isolation and preservation of the right testicular vessels and the ductus deferens. Appendectomy was performed to avoid the risk of infection and the need for subsequent surgery. The abdominal wall reconstruction entailed an extensive preperitoneal dissection from the midline incision, extending laterally on both sides. To relieve pressure and enhance mobility, a transversus abdominis release was conducted. The inferior epigastric vessels were preserved on both sides. In the cranial direction, the preperitoneal dissection was performed to a depth well below the xiphoid and ribs, and in the caudal direction, it reached a depth that exceeded the symphysis. Subsequently, the preperitoneal dissection extended laterally to the iliac vessels and kidneys, thereby preserving both spermatic cords. This resulted in the creation of a substantial mesh space and the mobilization of the abdominal components. The peritoneum was then closed medially. In total, three non-absorbable meshes were placed in the preperitoneal space in a tension-free manner. The initial mesh (Parietex 15x10 cm) (Minneapolis, MN: Medtronic) was situated within the right inguinal canal, serving to enclose the hernia.

In order to achieve double coverage and additional reinforcement, a second, larger mesh (Dynamesh IPOM 45x30 cm) (Aachen, Germany: DynaMesh) was placed behind the first one, covering the entire right abdomen, extending from the xiphoid to below the symphysis. Due to the patient's size, a third mesh (Dynamesh IPOM 30x20 cm) was placed to cover the left abdomen, including the reduced small left inguinal hernia. On both sides, meshes were employed to cover the inguinal and femoral canals and were secured around the ductus deferens with the objective of preventing the recurrence of the hernia. The meshes were affixed to one another with Prolene and fibrin glue (Tisseel; Deerfield, IL: Baxter). Subsequently, the fascia was closed in the midline with PDS loops (Raritan, NJ: Ethicon), and the meshes were again secured through this median suture. To alleviate pressure, the ventral fascia was incised longitudinally, in accordance with the Ramirez procedure. The skin on the abdomen and right thigh was closed after the placement of subcutaneous drains. Following the application of sterile draping to the abdomen, we proceeded with the scrotum. The skin was macerated and the scrotum was observed to be inflamed, with evidence of severe subcutaneous edema.

**Figure 4:**
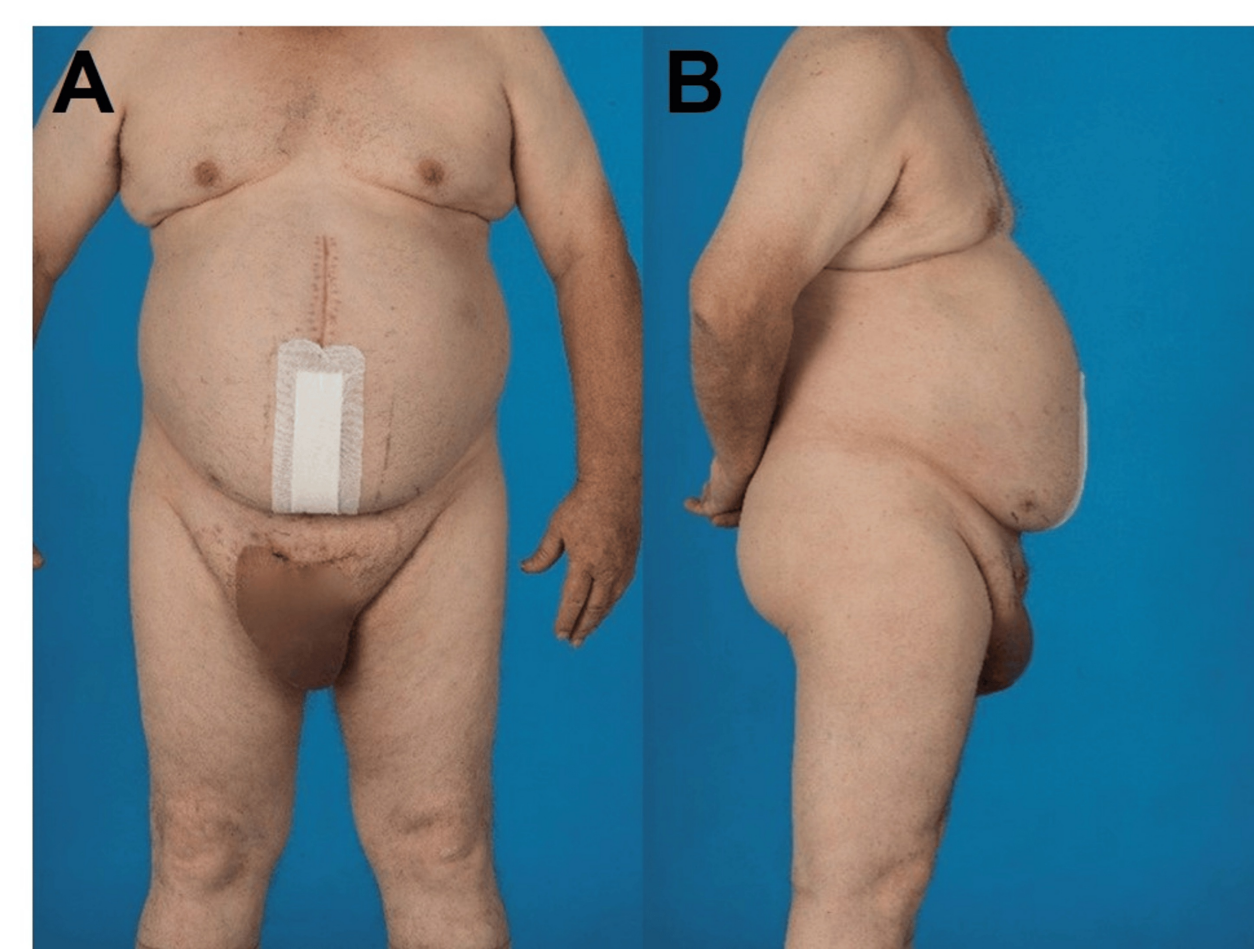
Postoperative result after successful hernia repair. (A) Postoperative image front view and (B) postoperative image lateral view.

Consequently, a significant quantity of scrotal skin was excised. The right testicle was of reduced size, with an adjacent large hydrocele. Although the right testicular vessels were preserved, perfusion was borderline. Given the aforementioned circumstances and the extensive nature of the wound and scrotal edema, we elected to proceed with the placement of a subcutaneous vacuum sponge and a second surgical procedure. A flexible abdominal belt was placed to prevent the formation of subcutaneous seroma. The surgical procedure lasted for a total of 430 min and the intraoperative course was free of complications. The patient was extubated immediately following the surgical procedure and was able to mobilize from the first postoperative day onwards. A gastric tube was placed intraoperatively to prevent postoperative paralysis and could be removed after two days. The perioperative thrombosis prophylaxis was administered in accordance with the standard protocol of our center, using unfractionated heparin (200 IE/kg continuous infusion over 24 h), commencing at six hours post-surgery. The oral diet was well tolerated and without postoperative paralysis. On the third postoperative day, the patient experienced an acute onset of dyspnea. CT scan revealed the presence of a pulmonary embolism and therapeutic anticoagulation was initiated, starting with an increase in the dose of unfractionated heparin (until anti-Xa activity exceeded 0.3 IU/mL). Before the patient was finally discharged, he was switched to apixaban (Eliquis) 5 mg twice daily. On the fourth postoperative day, a second-look surgery of the scrotum was performed. Subsequently, the right scrotum was determined to be non-viable due to ischemia and thus required removal. However, the edema had almost completely regressed, allowing the scrotal skin to be closed after drain placement. The subsequent course was uneventful, with wounds healing without problems, even on the scrotum. The patient was discharged after a total of 14 days and further follow-ups were uneventful. A subcutaneous seroma below the midline incision appeared without infection and was treated conservatively. The patient was kept on Eliquis 5 mg twice daily lifelong. Figures 4A, 4B show the postoperative result two months after surgery.

## Discussion

GIH with LOD poses a particular challenge in surgical and postoperative care, with a high prevalence of morbidity and mortality [9,10]. The greater the degree of LOD, the higher the risk of hernia reduction due to increased intra-abdominal pressure. Our case represents an extreme example of LOD, with the majority of the intestine herniated, necessitating special considerations in terms of surgical access, mesh placement, and wound management. The outcome was excellent, with no major surgical complications and excellent cosmetic and functional results. Despite the complexity of the surgical technique and the necessity for rigorous postoperative management of GIH and LOD, there is currently a paucity of established guidelines in this area. The primary concern is the loss of abdominal space, which can result in complications such as CR problems and ACS when attempting to reduce a hernia. Trakarnsagna et al. proposed a novel classification system for GIH with LOD and provided recommendations for the reduction of such hernias [9]. Type I GIH extends until the midpoint of the inner thigh, thus allowing for forced reduction to be a feasible option. In cases of type II and III GIH, which extend until the superior patella or below, the potential risks of forced reduction are too high, and the procedure is contraindicated due to the increased likelihood of ACS and CR complications. In cases of massive hernia content, surgical resection may be required. The resection of hernia components may involve the greater omentum or even portions of the intestine. Nevertheless, this may potentially increase the risk of surgical complications, due to anastomotic failure or infection. It is for this reason that we consider the prevention and preoperative preparation of the patient to be of utmost importance. The patient's GIH was, however, of a considerable magnitude, corresponding to a type III. Furthermore, the LOD was excessive, with almost the entire viscera trapped in the hernia sac for an extended period. To complicate, the scrotum was severely affected, presenting with macerated skin and ipsilateral testicular atrophy. In addition, an umbilical and left-sided inguinal hernia were present. This necessitated the utilization of an extended surgical approach, combining inguinal access with median laparotomy, to facilitate hernia reduction and create sufficient abdominal space.

Meticulous preoperative preparation of the patient is needed to reduce surgical risk factors and avoid postoperative morbidity. A preoperative CT scan is essential for the accurate assessment of the hernia and LOD extent, thus enabling the selection of the optimal surgical access route. In this particular case, the extensive nature of the hernia necessitated an open surgical approach as the sole viable option. Furthermore, the considerable LOD volume required a midline laparotomy to facilitate a component separation technique, ensuring sufficient intra-abdominal space for hernia reduction.

A specific challenge in the management of GIH is the loss of domain. This phenomenon occurs as a result of the abdominal cavity having adapted to a state of emptiness over an extended period of time. As a result, the replacement of the herniated viscera can precipitate a sudden increase in intra-abdominal and intrathoracic pressures, which may in turn give rise to respiratory complications and high mortality rates. Techniques to address this problem include debulking of the abdominal contents or expansion of the abdominal cavity. Extensive bowel resection (total or hemicolectomy), omentectomy, splenectomy, and even small bowel resection have been documented as procedures that can be employed in this context [6]. However, these are highly invasive and carry the risk of anastomotic insufficiency, which may lead to peritonitis and sepsis [10].

In recent years, the use of botulinum toxin A (BTX) has grown in popularity, becoming an increasingly prominent technique, or as an alternative to other established procedures [11]. Injection of BTX into the muscles of the abdominal wall results in the temporary paralysis of those muscles. This reduction in muscle tension facilitates the repair of hernias and allows for a reduction in the presence of massive LOD. The peak effect of BTX is observed two to four weeks after application [11,12]. This technique was applied in our patient three weeks before surgery, with a positive outcome. However, the true effect of this technique is difficult to evaluate, particularly given that a median laparotomy with component separation has been performed. Nevertheless, we recommend the additional application of BTX when other risk factors, such as obesity and pulmonary disease, are present, to minimize the risk of CR complications or ACS.

A further innovative approach for complex ventral hernias is the technique of posterior component separation with transversus abdominis release (TAR) [7]. This involves incising the posterior rectus sheath and developing the retrorectus plane laterally to the rectus sheath. The transversus abdominis muscle is dissected medially to the semilunar line, thereby exposing a wide plane while ensuring the preservation of the neurovascular bundles. The posterior rectus fascia is advanced medially, and the mesh is placed in a sublayer fashion with the linea alba restored ventral to the mesh [7,13]. This approach is safe, effective, and reliable, with low perioperative morbidity and recurrence rates. It is also associated with a low risk of skin necrosis and surgical site infection [8].

An alternative approach to creating additional space prior to the reduction of giant hernias is the utilization of preoperative pneumoperitoneum. The concept of progressive preoperative pneumoperitoneum (PPP) was first introduced in the 1940s by Moreno [14]. This technique allows for the relaxation of the abdominal wall through the gradual distension of the retracted abdominal wall muscles. It can be beneficial in cases of LOD, as it increases abdominal capacity and facilitates intestinal reintegration and lung adaptation after reduction. However, it should be noted that the use of this technique requires a prolonged hospital stay, and there is a risk that the insufflated gas can enlarge the hernia instead of expanding the abdominal space. Hence, this technique is no longer used routinely.

There are several possible surgical approaches. The abdominal and inguinal approaches can be utilized in combination when necessary [10]. Our surgical technique corresponds to an extended Stoppa approach, initially described by Stoppa [15]. The procedure entails the placement of a mesh within the preperitoneal space, thereby minimizing the impairment of muscular and fascial vascularization. This is followed by the closure of the anterior fascia. Over time, this mesh position has become the standard technique for complex incisional hernias, with remarkably low recurrence rates for incisional hernias. Furthermore, the preperitoneal position of the mesh limits the formation of adhesions with the bowel. The combination of this approach with a technique for separating abdominal components allows for the creation of space and the implementation of a tension-free hernia repair, thereby offering considerable advantages. The technique of abdominal component separation was first described by Ramirez et al. in 1990 [5].

As anticipated from the CT scan, most of the intestine - except for the proximal jejunum, transverse colon, and left-sided colon - had herniated through the right inguinal canal. Although the bowel was compressed at its passage, there were no indications of strangulation or ileus, which is consistent with the patient's relatively mild symptoms. Nevertheless, reduction from the inside was not feasible due to the narrow passageway. It was thus decided that a second incision should be made below the inguinal canal along the ventral aspect of the thigh. This enabled the contents of the hernia to be dissected freely and resection of the hernia sac to be performed. Subsequently, the hernia was completely reduced by pressure from both sides, abdominal and inguinal. However, even with our combined transabdominal and inguinal open access, the reduction was complex, and excessive force or tear could easily have caused intestinal perforation or ischemia.

In this case, a laparoscopic approach would not have been feasible due to the large amount of intestine in the hernia sac, which was both trapped and not strangulated, posing a particular challenge in hernia reduction. Furthermore, the patient's obesity and pulmonary comorbidities would have made laparoscopic access with a prolonged head-down position more difficult.

## Conclusions

Surgical repair of GIH with excessive LOD poses a particular challenge, due to the potential risks of ACS and CR complications. In the case of GIH with LOD, we recommend an open surgical approach via midline laparotomy. This facilitates hernia reduction and allows for abdominal component separation technique and preperitoneal mesh placement. This minimizes the risk of recurrence while enabling the best results in terms of cosmetics, functionality, and abdominal wall integrity.
